# Translocation of bacterial LPS is associated with self-reported cognitive abilities in men living with HIV receiving antiretroviral therapy

**DOI:** 10.1186/s12981-023-00525-z

**Published:** 2023-05-18

**Authors:** Stéphane Isnard, Léna Royston, Susan C. Scott, Tsoarello Mabanga, John Lin, Brandon Fombuena, Simeng Bu, Carolina A. Berini, Mark S. Goldberg, Malcolm Finkelman, Marie-Josée Brouillette, Lesley K. Fellows, Nancy E. Mayo, Jean-Pierre Routy

**Affiliations:** 1 Infectious Disease and Immunity in Global Health Program, Research Institute of McGill University Health Centre, 1001 Boulevard Décarie, Montreal, QC H4A 3J1 Canada; 2 Chronic Viral Illness Service, McGill University Health Centre, Montreal, QC Canada; 3 CIHR Canadian HIV Trials Network, Vancouver, BC Canada; 4 Division of Infectious Diseases, Geneva University Hospitals, Geneva, Switzerland; 5 Division of Clinical Epidemiology, Center for Outcomes Research and Evaluation, McGill University Health Centre (MUHC), Montreal, Canada; 6 Department of Medicine, McGill University, Montreal, Canada; 7 Associates of Cape Cod Inc, Falmouth, MA USA; 8 Department of Psychiatry, McGill University, Montreal, Canada; 9 Department of Neurology & Neurosurgery, Montreal Neurological Institute, McGill University, Montreal, Canada; 10 School of Physical and Occupational Therapy, McGill University, Montreal, Canada; 11 Department of Medicine, Division of Geriatrics, McGill University, Montreal, Canada; 12 Division of Hematology, McGill University Health Centre, Montreal, QC Canada

**Keywords:** People living with HIV, Cognition, Gut, Microbial translocation, LPS

## Abstract

**Background:**

Gut damage allows translocation of bacterial lipopolysaccharide (LPS) and fungal β-D-glucan (BDG) into the blood. This microbial translocation contributes to systemic inflammation and risk of non-AIDS comorbidities in people living with HIV, including those receiving antiretroviral therapy (ART). We assessed whether markers of gut damage and microbial translocation were associated with cognition in ART-treated PLWH.

**Methods:**

Eighty ART-treated men living with HIV from the Positive Brain Health Now Canadian cohort were included. Brief cognitive ability measure (B-CAM) and 20-item patient deficit questionnaire (PDQ) were administered to all participants. Three groups were selected based on their B-CAM levels. We excluded participants who received proton pump inhibitors or antiacids in the past 3 months. Cannabis users were also excluded. Plasma levels of intestinal fatty acid binding protein (I-FABP), regenerating islet-derived protein 3 α (REG3α), and lipopolysaccharides (LPS = were quantified by ELISA, while 1–3-β-D-glucan BDG) levels were assessed using the Fungitell assay. Univariable, multivariable, and splines analyses were performed.

**Results:**

Plasma levels of I-FABP, REG3α, LPS and BDG were not different between groups of low, intermediate and high B-CAM levels. However, LPS and REG3α levels were higher in participants with PDQ higher than the median.

Multivariable analyses showed that LPS association with PDQ, but not B-CAM, was independent of age and level of education. I-FABP, REG3α, and BDG levels were not associated with B-CAM nor PDQ levels in multivariable analyses.

**Conclusion:**

In this well characterized cohort of ART-treated men living with HIV, bacterial but not fungal translocation was associated with presence of cognitive difficulties. These results need replication in larger samples.

**Supplementary Information:**

The online version contains supplementary material available at 10.1186/s12981-023-00525-z.

## Background

The gut mucosa plays key roles in physiology and health. By targeting mucosal CD4 T-cells, HIV profoundly affects gut homeostasis, promotes epithelial cell death and decreases cell–cell adhesion. Circulating markers of gut integrity have been used to characterized levels of leaky gut in people living with HIV (PLWH). Intestinal fatty acid-binding protein (I-FABP) is released in the circulation upon gut epithelium cell death. I-FABP blood levels have been found elevated in PLWH taking or not antiretroviral therapy (ART) compared to control without HIV in numerous studies. Higher levels of regenerating islet-derived protein 3 α have also been found in PLWH, regardless of their use of ART, compared to controls [1]. REG3α is an antimicrobial peptide produced in the gut lumen. Upon gut damage, REG3α translocate in the submucosa and its presence in the circulation reflects gut permeability.

HIV-associated gut damage allows passage of microbial products in the submucosa and systemic circulation. This microbial translocation is commonly characterized by quantification of bacterial product lipopolysaccharide (LPS) in blood. Indeed, LPS levels have been shown elevated in both ART-naïve and ART-treated PLWH. Translocation of fungal products β-D-glucan (BDG) has also been described during HIV infection and elevated blood levels of BDG persist in ART-treated PLWH [2, 3]. Both bacterial and fungal products have been shown to participate in persisting levels of inflammation and increased risk of non-AIDS comorbidities in ART-treated PLWH [2, 4–6].

Gut damage and microbial translocation have been recurrently associated with inflammation and increased risk of non-AIDS comorbidities in ART-treated PLWH such as adiposity [7], cardiopulmonary function [3], and neurocognitive impairment [8–10]. LPS levels have been associated with HIV-associated neurocognitive disorder (HAND), especially in PLWH with hepatitis C infection [11], and with slower processing speed in suppressed alcohol heavy drinkers [12]. Circulating BDG levels have been associated with neurocognitive performance assessed with Global Deficit Score (GDS) [8, 9].

Markers of gut damage, bacterial and fungal translocations have not been compared in their association with neurocognitive performance in ART-treated PLWH. We performed such comparisons in a well characterized group of ART-treated men living with HIV. Cognition was assessed in two ways: performance-based cognitive ability was quantified using the B-CAM [13]; and self-reported cognitive difficulties were documented with the 20-item Patient Deficit Questionnaire (PDQ) that assess memory (retrospective and prospective), attention, organization and planning over the previous 4 weeks.

## Methods

### Study design and population

In a cross-sectional analysis, a total of 80 ART-treated men living with HIV (MLWH) were selected from the Positive Brain Health Now (CIHR/CTN 372) cohort. The study design and protocol of the study have been reported [14, 15]. Samples were drawn from all participants who completed Visit 1, had available measures of cognition (performance-based and self-report) and frozen biological samples, after excluding participants (i) using anti-acids or proton pump inhibitors as those drugs modulate inflammation; and (ii) with high alcohol use (six or more drinks on one occasion more than once per month or more than 14 standard drinks/week) or cannabis use in the past 3 months. Participants with known inflammatory bowel diseases were also excluded. Current smoking was also excluded at selection, but due to additions to the data after the sample was drawn, 4 people were in fact current smokers. Men were selected as they represent the largest group and the association between inflammation and cognition could vary by sex [16, 17]. Blood collection was performed in the morning in fasting participants.

Participants were classified into three different groups (around 26–27 per group) based on an earlier version of B-CAM score at baseline (low [20–56], intermediate [58–68] or high [70–100]).

### Cognitive assessment

To assess performance based self reported cognition, we used B-CAM, a computerized measure of cognition that yields a continuous score. The B-CAM consists of a series of tests including Corsi block task (forward and backward), Eriksen flanker task-incongruent reaction time, mini Trail Making test B (as in the Montreal Cognitive Assessment (MoCA), letter fluency (F-A-S in English, P-L-S in French, 1 min) and word list learning with recall of 8 words [13, 18]. The scoring range of the version available from 0 to 100, with higher values indicating better cognitive ability, with a standard deviation (SD) of 15 (when transformed from the original scale [19]). This test is administered at all visits in the Positive Brain Health Now study.

Presence of cognitive difficulties was documented using the self-reported 20-item Patient Deficit Questionnaire (PDQ), which assesses self-reported memory, attention, organization, and planning over the previous 4 weeks. The questionnaire contains 20 items and leads to a total score on 80, with a SD of 17.5 [19]. A higher score is associated with more cognitive difficulties. The questionnaire pertains to everyday activities of interest to clinicians, such as adherence to care (e.g., “I forget to take medication” or “I forget medical appointments”) and safety (e.g., “I forget to turn off the stove”) and elicits cognitive difficulties that are frequent among PLWH (e.g., “trouble with concentration”). Importantly, the PDQ is brief and can be successfully completed by people with mild to moderate cognitive impairment [20].

### Laboratory measurements

HIV infection was diagnosed by quantifying HIV-1 p24 antigen/antibody in plasma and confirmed by Western blot as previously reported [21]. Plasma viral load was quantified by the Abbott RealTime HIV-1 assay (Abbott Laboratories). Plasma samples of study participants were drawn fasting and stored at − 80 °C until used. CD4 and CD8 T-cell counts were measured using 4-color flow cytometry [22]. Peripheral blood mononuclear cell samples of study participants were stored in liquid nitrogen until used.

### Measurement of markers of epithelial gut damage and microbial translocation

I-FABP, REG3α and LPS were measured by ELISA as previously described [1, 23]. Plasma BDG was measured by the Fungitell assay in duplicate as per the manufacturer’s instructions with lower extended range (as low as 3.9 pg/mL) (Associates of Cape Cod, Inc East Falmouth, MA).

### Statistical analyses

Medians with interquartile range were calculated for all continuous variables. Unpaired comparisons were conducted using *t* tests or Mann–Whitney's *U* tests. Spearman's rank correlation test identified associations between two quantitative measures. The Kruskal–Wallis’s test was used to compare more than 2 study groups. *P* values < 0.05 were considered significant. To control for potential confounding by classical neurocognitive risk factors on the association between each factor and neurocognition indexes, we performed multivariable linear regression analyses using SAS statistical software. Strength of associations were categorized based on effect sizes where β is the numerator and the SD of the B-CAM and PDQ in the entire cohort is the denominator. Cohen’ effect sizes ≤ 0.2 are considered small, effect sizes around 0.5 are considered medium and large is ≥ 0.8. GraphPad Prism 8.0 (GraphPad Software), SAS and R-software were used to perform descriptive statistical analyses. Splines were generated using R.

### Patient consent statement

This study was approved by the research ethics board of each participating institutions, including the McGill University Health Centre. Study participants provided written informed consent for study enrollment and participation. All matters were conducted in accordance with the principals of the Declaration of Helsinki.

## Results

### Characteristics of study participants

As shown in Table 1, all participants were male, living with HIV, and receiving ART. Their HIV viral load was below or equal 50 copies/mL. Their median age was 52. Participants received ART for an average of 12 years (6.6–17.8). Approximatively 36.2% of participants were non-white, and 80% had a post-secondary education level. Fifty-four percent had never smoked (Table 1).

**Table 1:** Participant characteristics

	Median	Interquartile range
Personal characteristics		
Age	52	46–59
Ethnicity		
White	51 (63.8%)	
Black	8 (10.0%)	
Mixed	7 (8.8%)	
Other/missing	14 (17.5%)	
Education		
High school	16 (20%)	
College (CEGEP), technical or vocational diploma	20 (25%)	
University level	44 (55.0%)	
Tobacco use		
Never	43 (54%)	
Past only	31 (39%)	
Current	4 (5%)	
Uncertain/Missing	2 (2.6%)	
Disease characteristics and laboratory values		
Duration of infection (years)	13.8	8.7–23.0
CD4 T cell count (Cells/µL of blood)	589	410–802
CD8 T cell count (Cells/µL of blood)	750	560–1026
CD4:CD8 ratio	0.8	0.5–1.1
HIV Viral load (copies/mL)	< 50	Undetectable—< 50
Plasma I-FABP (pg/mL)	965.5	659.6–1377
Plasma REG3α (pg/mL)	4302.4	2921–6396
Plasma LPS (pg/mL)	47.1	32.8–79
Plasma BDG (pg/mL)	14.5	9.0–28
Brain health outcomes		
B-CAM (0–100, higher is better)	61.76	52.94–72.06
PDQ (0–80, higher is worse)	26.25	13.75–40.63

Interestingly, B-CAM and PDQ values did not correlate. Similarly, gut damage and microbial translocation markers did not correlate with each other [24]. Participants appear to have similar LPS, BDG and REG3α levels to those of ART-treated PLWH in our previous work [25]. Levels of I-FABP appear similar to the one of HIV-uninfected controls, although highly variable.

### Higher plasma levels of the gut permeability marker REG3α are associated with lower self-reported cognition in ART-treated men living with HIV

We first compared plasma levels of gut permeability markers among ART-treated MLWH with different levels of cognition.

**Fig. 1﻿:**
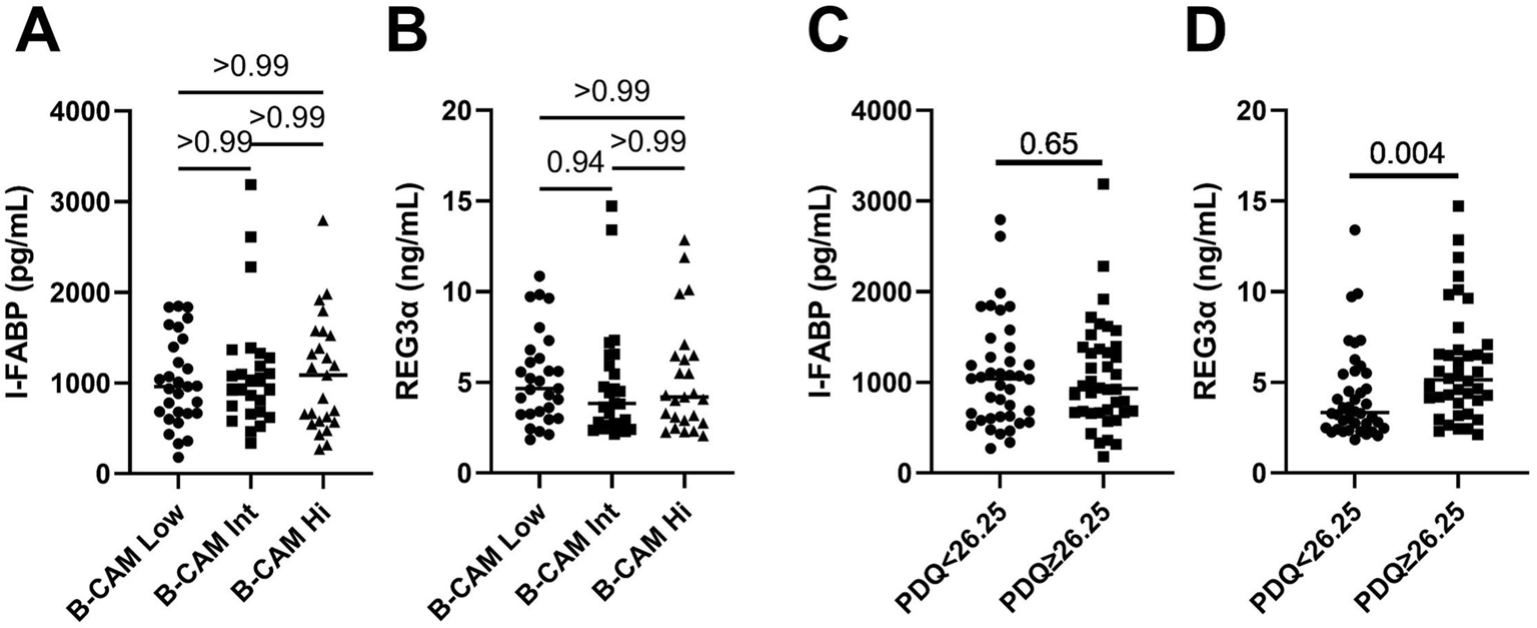
Levels of gut damage markers among groups of ART-treated men living with HIV with different cognitive function. Levels of the gut damage marker I-FABP (**A**, **C**) and gut permeability marker REG3α (**B**, **D**) in ART-treated men living with HIV with higher than the B-CAM groups (**A**, **B**) or with median PDQ lower or higher than the median of 26.25 (**C**, **D**). **A**, **B**: Kruskal–Wallis’s test with Dunn’s post-test. **C**, **D**: Mann Whitney’s test

When participants were grouped according to their B-CAM values (low [20–56], intermediate [58–68] or high [70–100]), although their levels tended to increase with worst outcome, no statistically significant differences in I-FABP (median 963, 979 and 1088 pg/mL respectively) or REG3α (4.7, 3.8 and 4.2 ng/mL respectively) levels were observed between groups (Fig. 1A, B).

When participants were grouped based on their PDQ levels, those with PDQ higher than the median 26.25 had similar levels of IFABP (1038 and 931 pg/mL respectively, p = 0.97) but higher levels of REG3α (3.3 and 5.1 ng/mL respectively, p = 0.004) (Fig. 1C, D).

### Microbial translocation of LPS is associated with low self-reported cognition with ART-treated men living with HIV

We then compared plasma levels of microbial translocation markers among ART-treated MLWH with different levels of cognition.

**Fig. 2:**
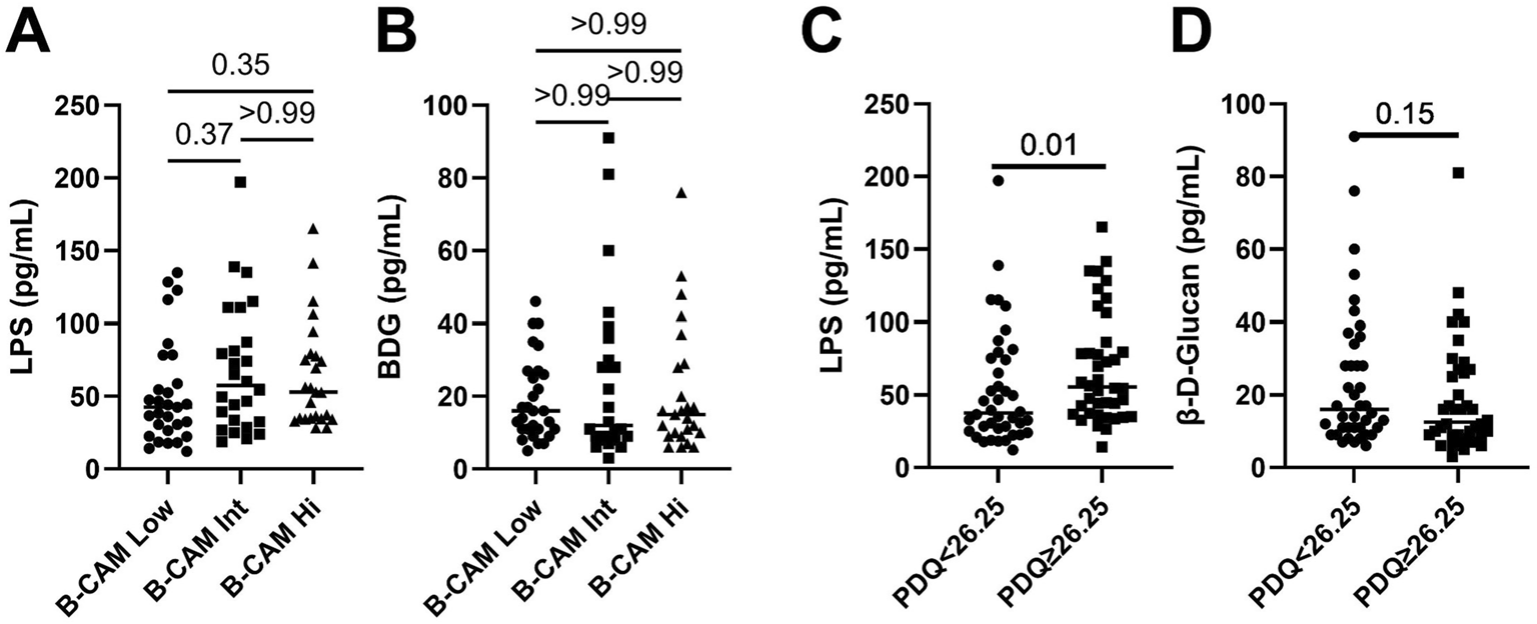
Levels of microbial translocation markers among groups of ART-treated men living with HIV with different cognitive function. Levels of the bacterial marker LPS (**A**, **C**) and fungal marker BDG (**B**, **D**) in ART-treated men living with HIV with higher than the B-CAM groups (A, B) or with median PDQ lower or higher than the median of 26.25 (**C**, **D**). **A**, **B**: Kruskal–Wallis’s test with Dunn’s post-test. **C**, **D**: Mann Whitney’s test. *LPS* Lipopolysaccharides, *BDG* 13-β-D-Glucan, *B-CAM* brief cognitive ability measure, *PDQ* patient deficit questionnaire

When participants were grouped according to their B-CAM values (low, intermediate or high), plasma levels of LPS (median 42.6, 57.5 and 53.0 pg/mL respectively) or BDG (16.0, 12.0 and 15.0 pg/mL respectively) were similar between groups (Fig. 2A, B).

When participants were grouped based on their PDQ levels, those with PDQ higher than the median 26.25 had higher levels of LPS (median 39.0 and 54.6 pg/mL respectively, p = 0.01) and similar levels of BDG (16.0 and 13.0 pg/mL respectively, p = 0.15) (Fig. 2C, D).

### Translocation of LPS is independently associated with low self-reported cognition

**Fig. 3:**
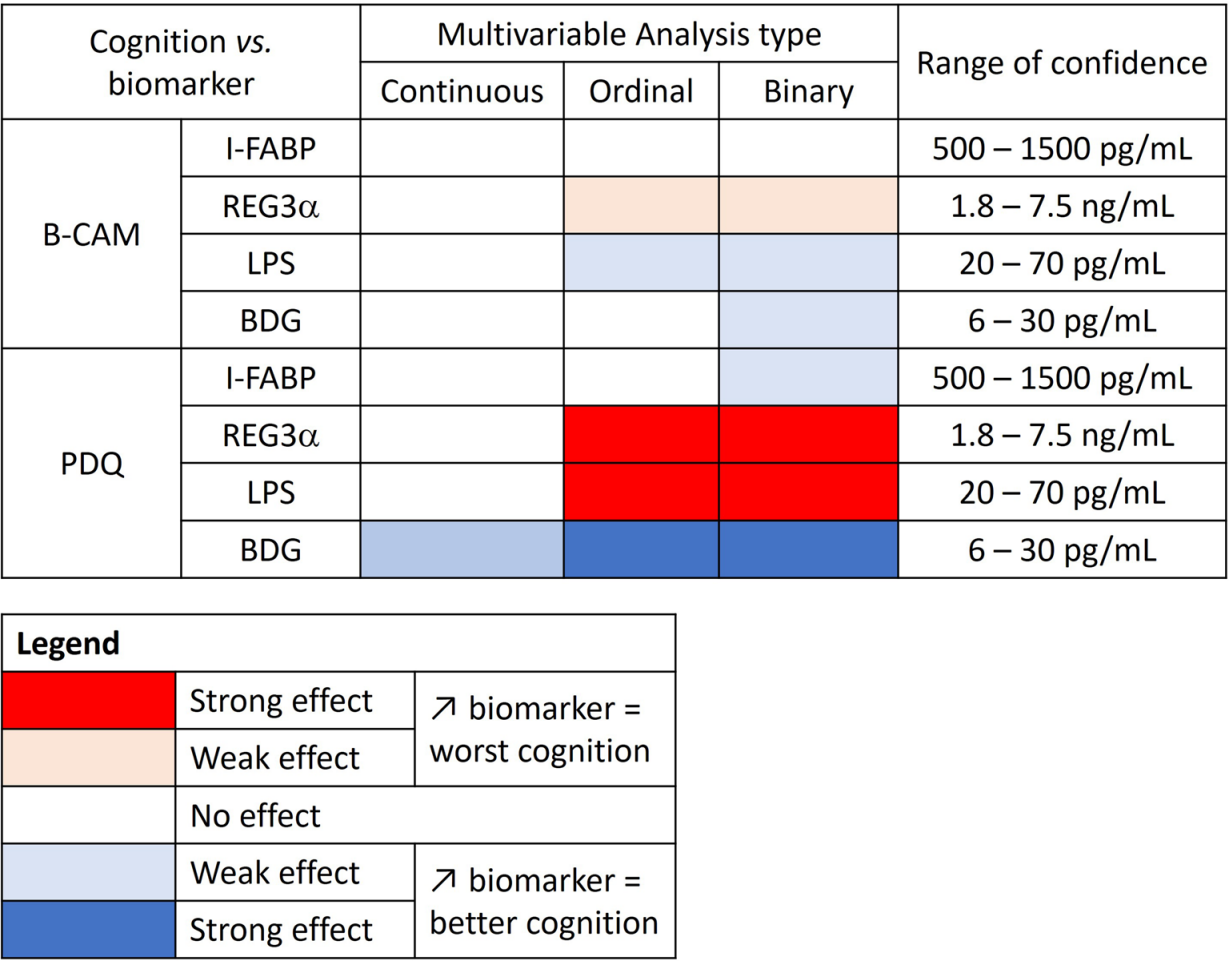
multivariable analyses of gut permeability and microbial translocation biomarkers with self-reported cognition. Influence of each cognition parameter on biomarkers levels is indicated with color levels. Cohen’ effect sizes ≤ 0.2 are considered small, effect sizes around 0.5 are considered medium and large is ≥ 0.8

We then performed multivariable analyses to assess the influence of known confounding factors on the association between gut markers and cognition by including age and education category in our models. Data were considered continuous, ordinal in 4 groups or binary. β was considered to assess the influence of each biomarker on cognition (Fig. 3).

When accounting for age and education, multivariable analyses showed only weak effects: REG3α seems to have a deleterious effect with increase in plasma levels associated with lower B-CAM values (negative β) when transformed into ordinal and binary data. LPS and BDG levels appeared to have weak protective effect, with plasma levels associated with higher B-CAM (positive β). None of those logistic regression analyses appeared statistically significant (Additional file 1: Table S1).

When looking at PDQ values, regression analyses showed that the association of LPS with worst self-reported cognition was independent of age and education category. Although a tendency was observed, higher levels of REG3α were not linked with lower cognitive abilities after adjustment (Fig. 3, Additional file 1: Table S1). Education but not age appeared to influence REG3α association with PDQ. Higher plasma BDG levels had a small effect on self-reported cognition (lower PDQ score), with an effect size of 0.27. However, the magnitude of the association was not statistically significant (Fig. 3, Additional file 1: Table S1).

None of the gut permeability markers correlated with CD4 T-cell count, and addition of CD4 T-cell count as a confounding variable to the multivariable analysis mildly changed R, β, and p-values, and did not influence our conclusion.

**Fig. 4:**
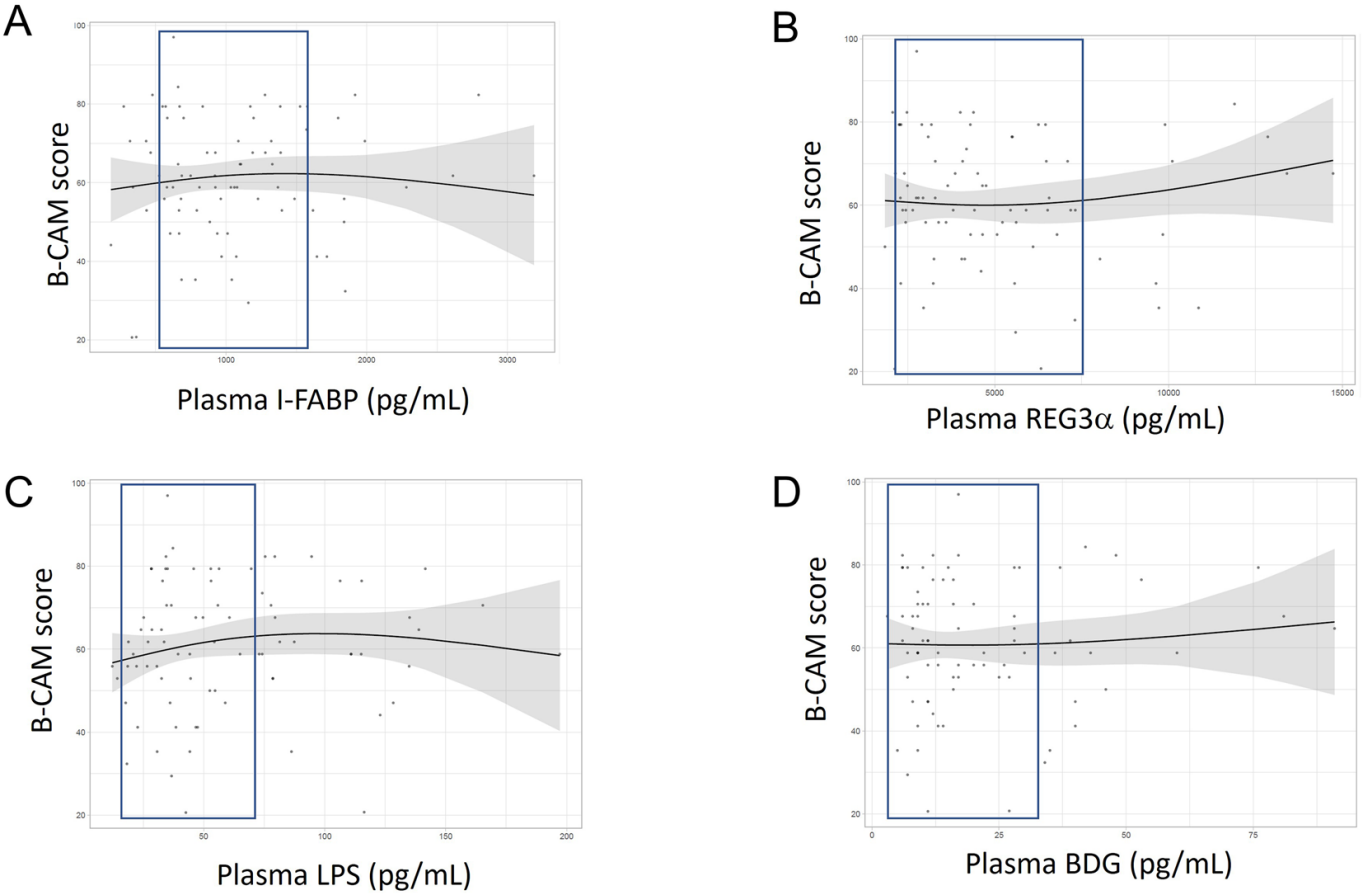
Spline graphs of gut damage markers vs. B-CAM adjusted for age and education category in men living with HIV. I-FABP (**A**), REG3α (**B**), LPS (**C**) or BDG (**D**) levels. Splines with 2 degrees of freedom are depicted. Confidence interval is shown in a blue frame

Splines analyses were performed for each association (Fig. 4). Confidence intervals were selected for each biomarker (Fig. 3). Between 20 and 70 pg/mL, LPS seemed to have a positive association with B-CAM, indicating that higher LPS levels were unexpectedly associated with better cognition (Fig. 4C).

**Fig. 5:**
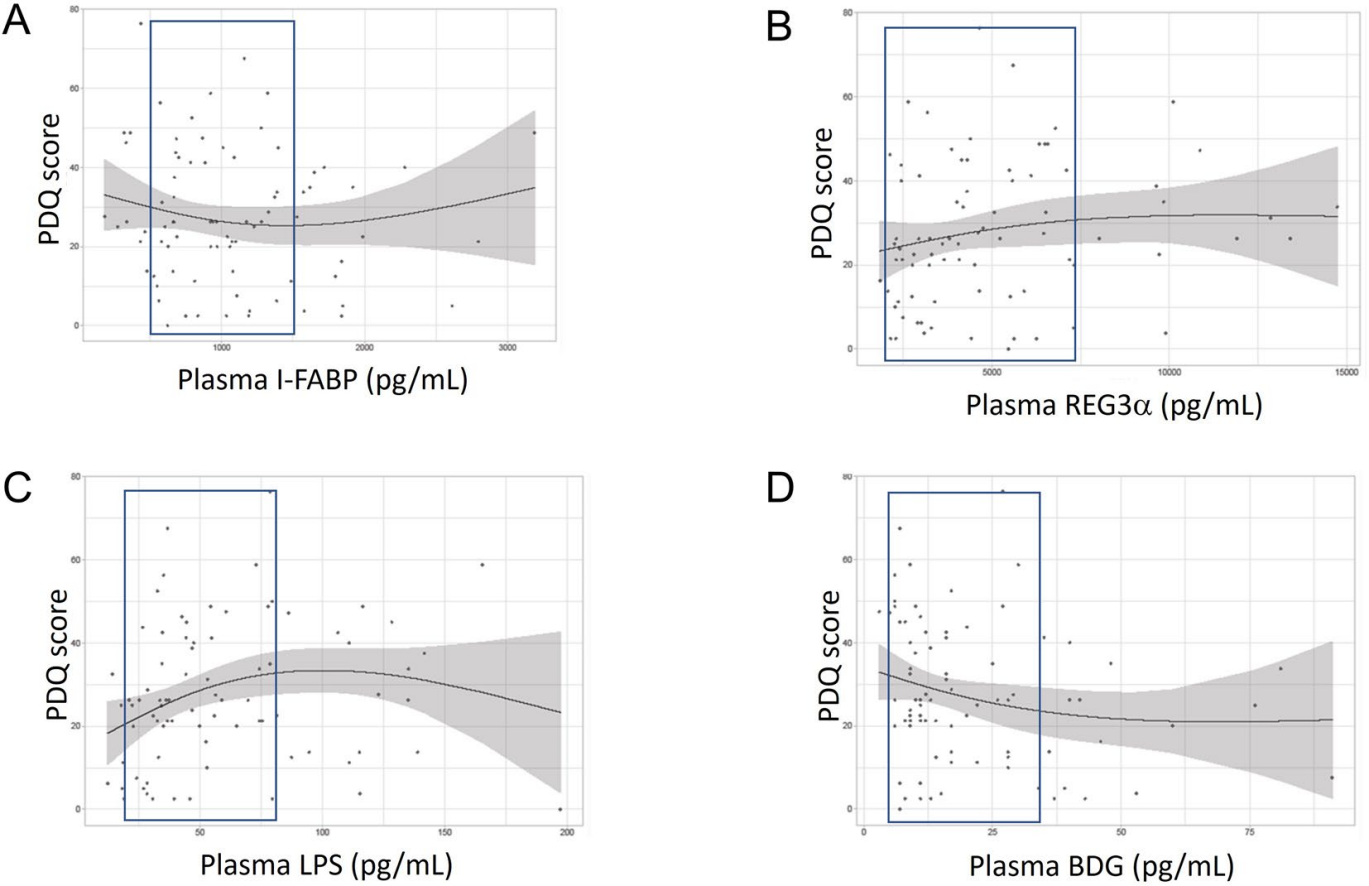
Spline graphs of gut damage markers vs. PDQ adjusted for age and education category in men living with HIV. I-FABP (**A**), REG3α (**B**), LPS (**C**) or BDG (**D**) levels. Splines with 2 degrees of freedom are depicted. Confidence interval is shown in a blue frame. *I-FABP* intestinal fatty acid binding protein, *REG3α* regenerating islet-derived protein 3 α, *B-CAM* brief cognitive ability measure, *PDQ* patient deficit questionnaire, *LPS* Lipopolysaccharides, *BDG* 1-3-β-D-Glucan, *B-CAM* brief cognitive ability measure, *PDQ* patient deficit questionnaire

Splines analyses of each biomarker and PDQ score showed that LPS appeared to have a strong link with worst cognition between 20 and 70 pg/mL (Fig. 5C). REG3α levels had a similar but weaker link with cognition (Fig. 5B). I-FABP and BDG levels had weaker protective effect (Fig. 5A and D).

## Discussions

Herein, we show that microbial translocation of bacterial LPS in the blood is associated with worst self-reported cognition in ART-treated MLWH, independent of age and education levels. Such association was observed using univariable, multivariable logistic and splines analyses. REG3α levels also showed such an association with self reported cognition, which was decreased after consideration of education categories. We did not find an association between self-reported cognitive function with levels of I-FABP or between any of the measured biomarkers of microbial translocation and performance-based cognitive ability. Interestingly, we have also previously shown that I-FABP levels were stable after 2 years on ART, when REG3α levels decreased, indicating that decreased REG3α but not I-FABP levels might indicate mucosal epithelium reparation. Hence, elevated REG3α levels could reflect a lack of gut repair, then being associated with chronic inflammation and decreased cognition [1].

BDG can be associated with stronger immune functions by trained immunity, a form of memory of the innate immune system allowing stronger responses to repeated stimuli. However, BDG stimulation of trained immunity in the context of chronic stimulation, such as the one we would expect from microbial translocation in PLWH, has not been described and still under debate [26, 27]. Hence, the link between BDG and self-reported cognition should be replicated in larger cohorts.

Our study is unique in considering both performance-based and self-reported cognition. While existing studies have found an association between a diagnosis of HAND and various biomarkers of microbial translocation, we are the first to report an association between markers of gut permeability and self-reported cognitive function. This finding is important because self-reported cognition is associated with everyday function and quality of life to a much greater extent than a diagnosis of HAND [28, 29], suggesting that microbial translocation is of clinical importance to outcomes that are meaningful for persons living with HIV.

A few reasons may explain the discrepancy between the findings of association with self-reported cognition versus the lack of association with performance-based cognition. First, there are two construct that, while overlapping, represent distinct aspects of cognition. A recent NIMH-sponsored meeting on biotypes of CNS complications in persons living with HIV highlighted the heterogeneity of manifestations of neuro-HIV [30] and the presence of self-reported cognitive difficulties may represent one phenotype of brain involvement. We did not test the association between biomarkers of gut permeability and a diagnosis of HAND for several reasons. This diagnosis, which required administration of neuropsychological testing, was not available in all participants; the diagnosis is prone to misclassification [28]; and continuous measures are more likely to uncover small associations. The B-CAM, which was developed specifically to measure cognition in persons living with HIV, has the strong psychometric properties required to maximize the likelihood of identifying relationships.

The source of microbial translocation is still debated in ART-treated PLWH. A consensus in forming toward microbial product found in blood being of gut origin. Moreover, the concurrent association of cognitive dysfunction with both the gut permeability marker REG3α and LPS are in accordance with this statement. We have showed that translocation markers are independent of food intake [31], although they vary during the day [23]. Daily variations of microbial translocation markers should be considered with time of blood collection and circadian rhythm of participants taken into account in future analyses.

Possible mechanisms of microbial translocation-associated decrease in cognitive abilities also lend support to the importance of inflammatory pathways. Interestingly, LPS [32] and BDG [9] have been found to cross the blood–brain barrier. There, they could induce inflammation, and participate in disorganisation of neuronal networks. Also, systemic levels of LPS and BDG induce inflammation [5, 12]. For instance, LPS activation of monocytes has been associated with dementia in AIDS patients [33]. Direct activation of cells or cells activated in an LPS-induced inflammatory environment have the capacity to cross the blood brain barrier and disrupt brain homeostasis. Finally, the gut microbiota could also play a role as dysbiosis has been implicated in disruption of gut homeostasis, gut permeability and inflammation. For instance, different abundance of the mucin-degrading *Akkermansia muciniphila* in the gut has been link with variations in brain functions through a gut-brain axis [34, 35]. Indeed, we have also observed that gut dysbiosis was linked with microbial translocation in PLWH [36]. Overall, microbial translocation-induced inflammation perturbs brain function, and persisting gut permeability on ART could be associated with brain inflammation and decreased cognitive abilities.

In addition to the cross-sectional nature of our studies, our study is limited by the lack of prospective data. Indeed, microbial translocation could have participated in deteriorating cognitive function, before partly resorbing. Also, we have shown that I-FABP and LPS plasma levels variate daily in ART-treated PLWH, time of blood collection and circadian rhythm of participants should be considered in further analyses [23]. Finally, the link between microbial translocation and cognitive abilities in women living with HIV will have to be assessed in further studies.

It is difficult to compare our results in HIV negative people as behaviour and lifestyle are different. Future studies could compare PLWH to HIV negative controls recruited in the same medical centres and sex-health clinics.

## Conclusion

Lower cognitive performance is still burdening PLWH even after years of ART. Persisting inflammation is responsible for the increased risk of non-AIDS comorbidities and accelerated aging in ART-treated PLWH, including for neurocognitive disorders. Our study findings suggest that gut permeability and microbial translocation are associated with the presence of self-reported cognitive difficulties in some PLWH. The findings need to be replicated and our estimates of effect size can be used to inform optimal sample sizes for future studies. The relationship between these biomarkers and changes in cognition should also be examined, as is the co-calibration between biomarkers values and cognition longitudinally. New therapeutic interventions are required to improve gut health in PLWH, reduce the risk of developing non-AIDS comorbidities to prevent cognitive disorders in ART-treated PLWH [37–40].

## Supplementary Information

**Additional file 1: Table S1**: Univariable and multivariable analyses.

## Data Availability

Data and materials are available upon reasonable request to corresponding author. Data were presented in part virtually at the AIDS 2022 conference in Montréal, QC, Canada.
