# Supplementary material for: Translocation of bacterial LPS is associated with self-reported cognitive abilities in men living with HIV receiving antiretroviral therapy
Source: AIDS Res Ther. 2023 May 18;20:30. doi: 10.1186/s12981-023-00525-z (PMC10193796; doi:10.1186/s12981-023-00525-z)
Supplement: Supplementary file 1 — Additional file 1: Table S1: Univariable and multivariable analyses. [file 12981_2023_525_MOESM1_ESM.docx]

**Additional file**

Table S1: Univariable and multivariable analyses

| B-CAM | | | | | | | | | | | |
| --- | --- | --- | --- | --- | --- | --- | --- | --- | --- | --- | --- |
|  |  | Univariate analysis | | | | | Adjusted for age and education category | | | Type of analysis | Effect size |
|  |  | R² | Βeta | | P value | | R² | Βeta | P value |  |  |
| I-FABP | Age, education | 0.0001 | 0.31 | | 0.93 | | 0.25 | -0.92 | 0.78 | Binary  0: 0-<965; 1: ≥965 | -0.0613 |
|  | + CD4 count | N/A | | | | | 0.24 | -0.63 | 0.85 |  |  |
| REG3α | Age, education | 0.037 | -6.3 | | 0.09 | | 0.25 | -2.73 | 0.42 | Binary  0: 0-<4.3; 1: ≥4.3 | -0.182 |
|  | + CD4 count | N/A | | | | | 0.24 | -0.44 | 0.69 |  |  |
| LPS | Age, education | 0.02 | 5.2 | | 0.20 | | 0.27 | 5.1 | 0.16 | Binary  0: 0 <33; 1: ≥33 | 0.34 |
|  | + CD4 count | N/A | | | | | 0.27 | 5.4 | 0.15 |  |  |
| BDG | Age, education | 0.002 | | -1.3 | | 0.73 | 0.25 | -0.45 | 0.89 | Binary  0: 0-20; 1: 20 | -0.03 |
|  | + CD4 count | N/A | | | | | 0.24 | -0.19 | 0.96 |  |  |

| PDQ | | | | | | | | | |
| --- | --- | --- | --- | --- | --- | --- | --- | --- | --- |
|  |  | Univariate analysis | | | Adjusted for age and education category | | | Type of analysis | Effect size |
|  |  | R² | Βeta | P value | R² | Βeta | P value |  |  |
| I-FABP | Age, education | 0.015 | -4.2 | 0.27 | 0.09 | -5.7 | 0.15 | Binary  0: 0-<965; 1: ≥965 | -0.33 |
|  | + CD4 count | N/A | | | 0.16 | -7.2 | 0.07 |  |  |
| REG3α | Age, education | 0.042 | 6.9 | 0.07 | 0.10 | 6.3 | 0.11 | Binary  0: 0-<4.3; 1: ≥4.3 | 0.36 |
|  | + CD4 count | N/A | | | 0.14 | 5.9 | 0.13 |  |  |
| LPS | Age, education | **0.094** | **11.9** | **0.006** | **0.17** | **12.9** | **0.003** | Binary  0: 0 <33; 1: ≥33 | **0.74** |
|  | + CD4 count | N/A | | | **0.22** | **13.1** | **0.003** |  |  |
| BDG | Age, education | 0.02 | -5.4 | 0.18 | 0.08 | -4.7 | 0.25 | Binary  0: 0-20; 1: ≥21 | 0.27 |
|  | + CD4 count | N/A | | | 0.13 | -4.5 | 0.28 |  |  |

Significant associations are indicated bolded.

CD4 T-cell were added in the model as categories (3= worst (<300 cells/µL); 2 (300 to 500 cells/µL); 1 (501 to 750 cells/µL) or 0 = best (>750 cells/µL).
